# Immunolocalization of the short neuropeptide F receptor in queen brains and ovaries of the red imported fire ant (*Solenopsis invicta *Buren)

**DOI:** 10.1186/1471-2202-12-57

**Published:** 2011-06-14

**Authors:** Hsiao-Ling Lu, Patricia V Pietrantonio

**Affiliations:** 1 Department of Entomology, Texas A&M University, College Station, TX 77843-2475, USA

## Abstract

**Background:**

Insect neuropeptides are involved in diverse physiological functions and can be released as neurotransmitters or neuromodulators acting within the central nervous system, and as circulating neurohormones in insect hemolymph. The insect short neuropeptide F (sNPF) peptides, related to the vertebrate neuropeptide Y (NPY) peptides, have been implicated in the regulation of food intake and body size, and play a gonadotropic role in the ovaries of some insect species. Recently the sNPF peptides were localized in the brain of larval and adult *Drosophila*. However, the location of the sNPF receptor, a G protein-coupled receptor (GPCR), has not yet been investigated in brains of any adult insect. To elucidate the sites of action of the sNPF peptide(s), the sNPF receptor tissue expression and cellular localization were analyzed in queens of the red imported fire ant, *Solenopsis invicta *Buren (Hymenoptera), an invasive social insect.

**Results:**

In the queen brains and subesophageal ganglion about 164 cells distributed in distinctive cell clusters (C1-C9 and C12) or as individual cells (C10, C11) were immuno-positive for the sNPF receptor. Most of these neurons are located in or near important sensory neuropils including the mushroom bodies, the antennal lobes, the central complex, and in different parts of the protocerebrum, as well as in the subesophageal ganglion. The localization of the sNPF receptor broadly links the receptor signaling pathway with circuits regulating learning and feeding behaviors. In ovaries from mated queens, the detection of sNPF receptor signal at the posterior end of oocytes in mid-oogenesis stage suggests that the sNPF signaling pathway may regulate processes at the oocyte pole.

**Conclusions:**

The analysis of sNPF receptor immunolocalization shows that the sNPF signaling cascade may be involved in diverse functions, and the sNPF peptide(s) may act in the brain as neurotransmitter(s) or neuromodulator(s), and in the ovaries as neurohormone(s). To our knowledge, this is the first report of the cellular localization of a sNPF receptor on the brain and ovaries of adult insects.

## Background

Information processing through neuronal networks in the central nervous system (CNS) is achieved through the release of neurotransmitters and/or neuromodulators from presynaptic neurons and the receiving of those signaling molecules by their respective receptors in the postsynaptic neurons. Additionally, the released neuromodulators can also diffuse and reach out to receptors located at nonsynaptic regions within the CNS. Neuropeptides are a complex group of signaling molecules which can act as neurotransmitters or neuromodulators within the CNS, and as circulating neurohormones in the hemolymph. In this way, neuropeptides influence numerous physiological processes in invertebrates [1]. Neuropeptides in the neuropeptide F (NPF) family have been identified in, or predicted from genomes of a broad range of invertebrate taxa, including insects; however, only few of the respective G protein-coupled receptors (GPCRs) have been identified or fully characterized [2-15]. Therefore, this study focuses on the immunolocalization of a short neuropeptide F (sNPF) receptor in the queen of the red important fire ant, *Solenopsis invicta *Buren (Hymenoptera: Formicidae).

Invertebrate NPF family neuropeptides are structurally and functionally related to the vertebrate neuropeptide Y (NPY) peptide family, which is involved in the regulation of feeding behavior, stress and obesity, blood pressure, anxiety, memory retention, and circadian rhythms [16-18]. The insect NPF family includes long and short NPF peptides [19]. The long NPF (referred to as "NPF") peptides range in size from 36 to 40 amino acid residues and the short NPF (sNPF) peptides range in size from 6 to 11 amino acid residues. Many studies on the long NPF signaling pathway concluded that it is involved in feeding and social behaviors, stress responses, and alcohol sedation sensitivity in the fruit fly *Drosophila melanogaster *[20-28], hindgut contraction in the blood-sucking bug *Rhodnius prolixus *[29], and in ovarian maturation in locusts [30]. In contrast, little information was available on the role of the sNPF signaling pathway until recent studies with *Drosophila*. Four *Drosophila *sNPF peptides (sNPF-1 to -4) are generated from the same sNPF precursor by enzymatic processing and modification. This sNPF peptide precursor was detected in about a thousand neurons in the CNS of 3^rd ^instar larvae, and in about five thousand neurons in the CNS of adults [1,31]. *Drosophila *gain-of-function mutants with sNPF overexpression in the nervous system display increased food intake, resulting in flies larger than the wild type, while loss-of-function mutants exhibit reduced food intake [32]. Further experiments have shown that the receptor for sNPF peptides, located in the insulin-producing median neurosecretory cells in *Drosophila *larvae brain, is the upstream regulator that controls the expression of insulin-like peptides through the activation of extracellular signal-related kinases (ERK) [33,34]. In other insects, sNPF signaling pathways also appear to be involved in feeding regulation. For example, in the fire ants, the sNPF receptor transcripts in the queen brain were decreased by starvation suggesting that the sNPF signaling cascade may play a role in feeding regulation [11]. Moreover, during diapause, the adult Colorado beetle (*Leptinotarsa decemlineata*) is devoid of sNPFs suggesting that the sNPFs might contribute to pre-diapause shifts in feeding behavior that lead to larger body size and reserve accumulation [35]. In the honey bee *Apis mellifera*, the sNPF and its receptor transcript expression levels were interpreted as associated with worker division of labor and feeding behavior [36,37]. The sNPF receptor transcript is up-regulated in brains of foragers under food-deprivation (without protein) suggesting that foragers are more sensitive to nutritional changes than nurses [37].

The sNPF signaling pathway appears to be involved in additional insect functions. For instance, the Colorado potato beetle sNPF peptide, Led-NPF-1, was shown to stimulate ovarian development in the locust *Locusta migratoria*, suggesting a potential gonadotropin role of the peptide [19,38]; however, it is not known if this is the peptide direct effect. Functions such as cardio-inhibitory activity on beetles [39], and an increase in locomotor behavior and the modulation of metabolic stress responses in the fruit fly [40,41] have also been discovered. In *Drosophila*, the diversity in the function of the sNPF pathway might be explained by the broad and abundant distribution of sNPF peptides discovered in the brain. In addition, the sNPF peptides have also been identified in the hemolymph of adult *Drosophila*, suggesting a potential neuroendocrine role [42]. However, the exact targets of the sNPF peptide in the adult insect CNS or other tissues are still unknown.

The sNPF receptor belongs to the GPCR Rhodopsin family and is an orthologue gene of the vertebrate NPY type 2 (Y2) receptor [43]. Insect sNPF receptors have been characterized from *S. invicta*, *D. melanogaster*, and the mosquito *Anopheles gambiae *[11,14,43,44]. Ligand-receptor binding assays of sNPF receptors from *D. melanogaster *and *A. gambiae *revealed that sNPF peptides that contain nine or more amino acids are more potent than those with eight or fewer amino acids [14,42,44,45]. *An. gambiae *sNPF-1 inhibited the production of forskolin-stimulated cAMP by sNPF receptor transfected cells, suggesting that the receptor may act via Gi/o signal pathway [14].

The fire ant sNPF receptor transcript is present in the brain, midgut, hindgut, Malpighian tubules, fat body, and ovaries of mated queens, as determined by RT-PCR; a particularly high level of receptor expression was detected in the brain [11]. The *Drosophila *sNPF receptor transcript is present in both central and peripheral nervous systems in larvae and adults [43,44]. The *An. gambiae *sNPF receptor transcript is also broadly expressed in different body parts, but the receptor protein is not detectable in the ovaries by western blot [14]. Studies in these three insect species clearly showed that sNPF receptor transcripts are expressed in different tissues. However, because of the presence of the sNPF receptor in the nervous system, the RT-PCR results for peripheral tissues shown in fire ants and *Drosophila *are not definitive to establish receptor tissue localization because the amplification could potentially arise from neuronal contamination. In addition, transcript presence may not be associated with receptor protein expression. Therefore, localization of the sNPF receptor is an important step in defining the functional sites of the sNPF. Importantly, to our knowledge, there is currently no report on sNPF receptor protein localization in the adult brain or ovaries of any insect, and the role of sNPF in ovarian development is still unknown. The only report is from *Drosophila *larvae brain in which the receptor protein is immunolocalized in a few median neurosecretory cells [33].

In the past five years, much effort has been made to characterize receptors for neuropeptides; however, there is still relatively little information regarding their distribution and function, and only a few receptors have been successfully localized in certain tissues using immunolabeling, or particularly in *Drosophila *by using transgenic flies (for reviews, see [1,12]). The role of the sNPF signaling pathway in essential physiological processes makes it an important subject for study in social insects, especially ants, in which the control of both feeding and reproduction is complex and poorly understood. To elucidate the role of sNPF signaling pathway in fire ants, we investigated the localization of the sNPF receptor in both the brain and the ovaries of queens. We present evidence that the sNPF peptide(s) not only function as neurotransmitter(s) or neuromodulator(s) within the brain, but also might act as neurohormone(s) targeting the ovaries.

## Results

### Expression of sNPF receptor in both brain and ovaries of fire ant queens

**Figure 1:**
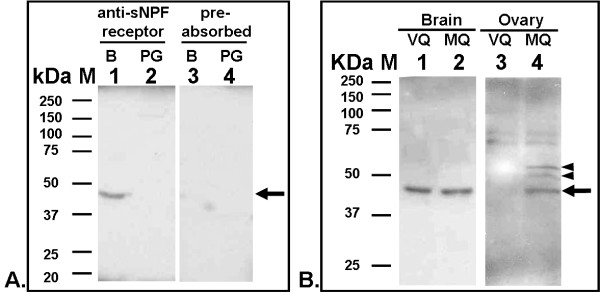
**Western blot analyses of the sNPF receptor expression in membranes from queens**. A: Membrane preparations (100 μg of protein per lane) of brains and subesophageal ganglion (SEG) (lanes 1 and 3, labeled with B) and postpharyngeal glands (lanes 2 and 4, labeled with PG) were analyzed with anti-sNPF receptor antibodies (lanes 1 and 2) or with antigen-preabsorbed antibodies (lanes 3 and 4). Only one band (~46.2 kDa) was specifically recognized in the membranes by the anti-sNPF receptor antibodies (lane 1, arrow), and no signal was detected using antigen-preabsorbed antibodies, as was expected (lane 3). No signal was detected in the membrane proteins from the postpharyngeal glands (lanes 2 and 4). B: Membrane proteins from brains and SEG (100 μg, lane 1 and 2) and ovaries (50 μg, lanes 3 and 4) of virgin queens (VQ) and mated queens (MQ) were also analyzed with anti-sNPF receptor antibodies. Similar receptor bands (~46.2 kDa) were detected in the membrane preparations of virgin and mated queens (lanes 1 and 2, arrow). The same size band (arrow) was also detected in mated queen ovaries (lane 4), but not in those of virgin queens (lane 3). In addition, two putative receptor bands (~55.3- and 51.1-kDa, lane 4, arrowheads) were detected in the mated queen ovaries but not in those of virgin queen ovaries (lane 3). M, marker.

To demonstrate the specificity of the antibodies developed against the fire ant sNPF receptor, we first performed western blot analyses of membrane proteins of queen brains, postpharyngeal glands, and ovaries (Figure 1). In the membrane proteins from brains of virgin queens, only one band was specifically recognized by the anti-sNPF receptor antibodies (Figure 1, A, lane 1). The estimated molecular weight (Mw) of the sNPF receptor band was ~46.2 kDa, corresponding to the predicted receptor Mw of 44.8 kDa. No signal was detected using antigen-preabsorbed antibodies, as expected (Figure 1, A, lane 3). Similar receptor expression levels were observed in the brains of virgin and mated queens (Figure 1, B, lanes 1 and 2). The postpharyngeal gland in the head of fire ant queen, which occupies a large portion of the head overlaying the brain, was used as a negative control tissue. No signal was detected using either anti-sNPF receptor antibodies (Figure 1, A, lane 2) or antigen-preabsorbed antibodies (Figure 1, A, lane 4), as expected. In the ovaries, three putative sNPF receptor bands (~46.2-, 51.1- and 55.3- kDa) were detected in the mated queen (Figure 1, B, lane 4), but not in the ovaries of virgin queens (Figure 1, B, lane 3). These different size bands in the mated queen ovaries are likely due to different post-translational modifications (including phosphorylation and N-glycosylation) predicted in the fire ant sNPF receptor protein sequence [11].

### Distribution of the sNPF receptor in the brains of fire ant queens

**Figure 2:**
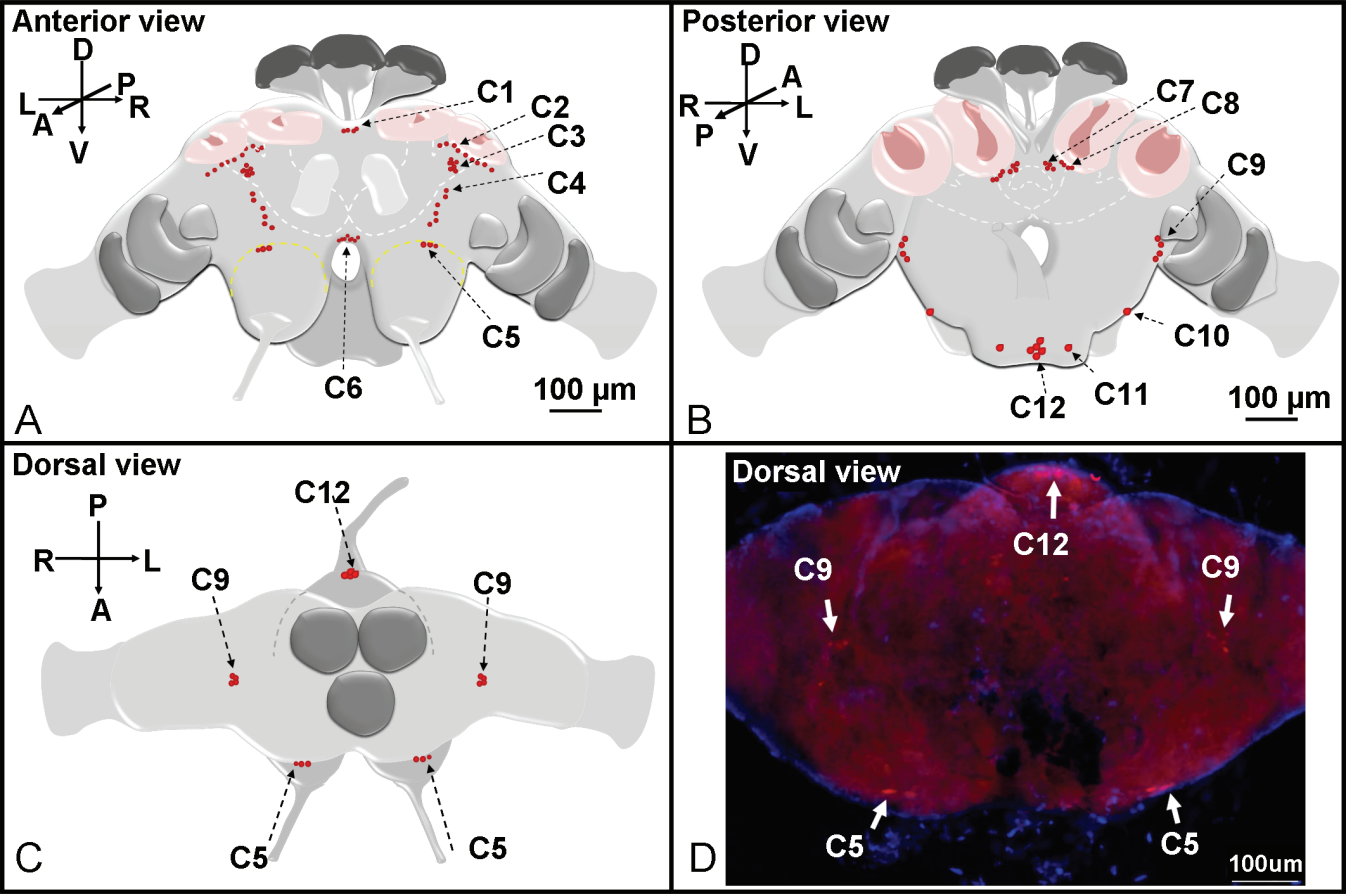
**Summary of the immunolocalization of the sNPF receptor in the queen brain and SEG**. A total of ~164 cells (shown as red dots) distributed in cell clusters (C1-C9 and C12) or as individual cells (C10, C11) were identified. Six cell clusters (C1-C6) are seen in anterior view (A) and C7-C12 (clusters or individual cells) are seen in the posterior view (B) of the queen brain and SEG. Dorsal views (C and D) show the relative positions of clusters C5, C9 and C12 cells. D: Merged image of pictures obtained with red (receptor signal) and blue (DAPI labeled in nuclei) filters.

**Figure 3:**
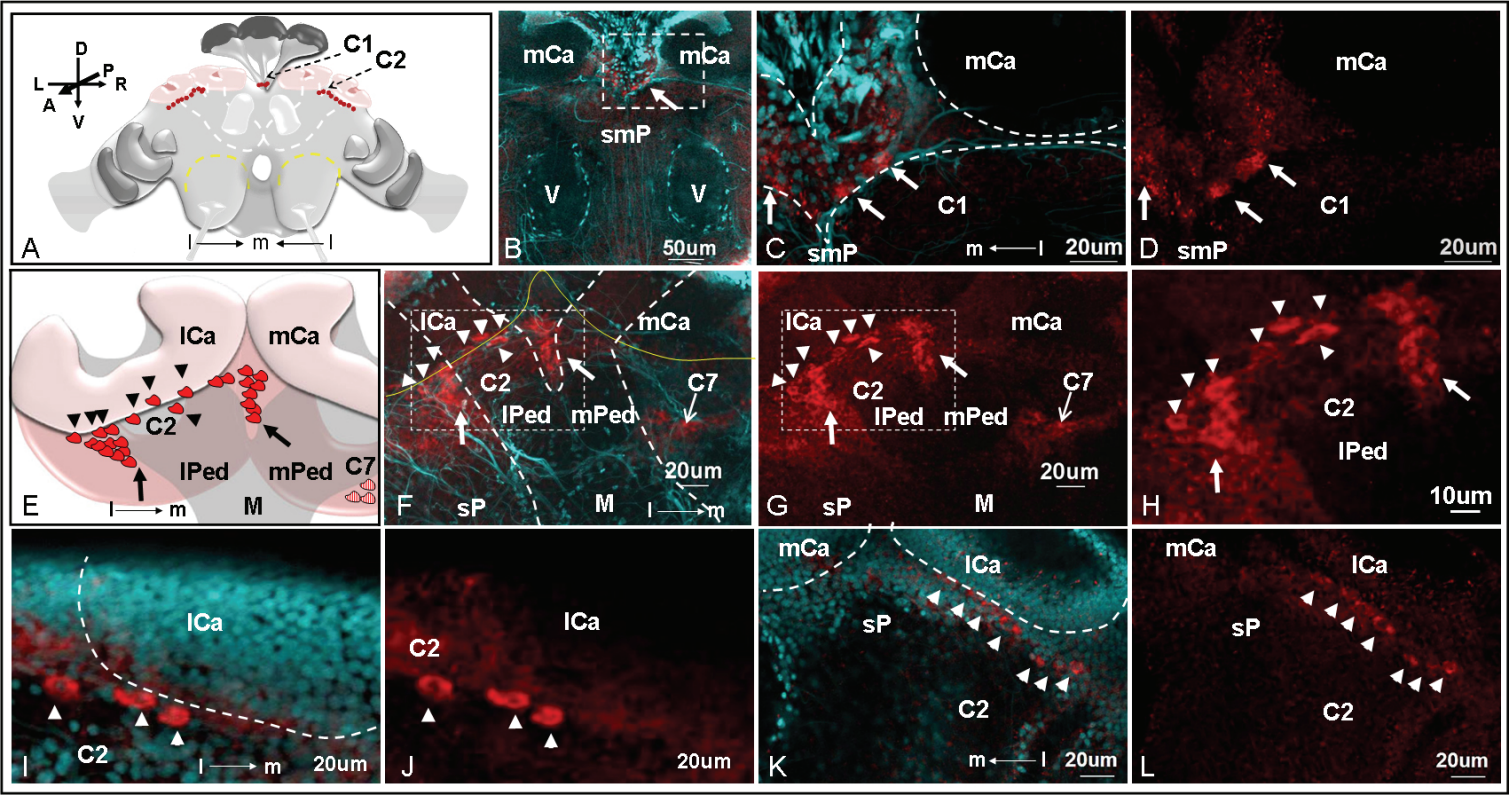
**Distribution of C1 and C2 sNPF receptor immunolabeled clusters observed in the anterior queen brain**. A: The position of cell clusters C1 and C2 in the anterior side of the brain are shown in a schematic. Confocal images obtained with single red channel for receptor signal (D, G, H, J and L) or red merged with cyan (DAPI nuclear stain) (B, C, F, I and K) are shown. B: Single image of cluster C1 (arrow) located in the superior medial protocerebrum (smP). V: vertical lobe of the mushroom body. C and D: Higher magnification images of B show that three cells (arrows) are detected with sNPF receptor antibodies. mCa: median calyces. E-L: Cluster C2 (~25 cells) is located near the lateral calyces (lCa) of the mushroom bodies in the superior protocerebrum. The cluster C7 closer to the posterior side of the brain (F and G, thin arrow), seen faintly through the section, will be described in figure 5. E: A schematic showing the relative position of C2 cells near the lCa. F and G: The cluster C2 cells surround the anterior half-side of the lateral pedunculi (lPed). H: Higher magnification image of G. I-L: Out of 25 cells in cluster C2, seven cells (arrowheads) located closer to the anterior surface of the lCa are likely lateral neurosecretory cells (arrowheads). Confocal stacked images thickness: 21.42 μm for C and D; 33.66 μm for F-H; 9.24 μm for K and L. M-P: Whole mount image obtained with Axioimager A1 microscope.

**Figure 4:**
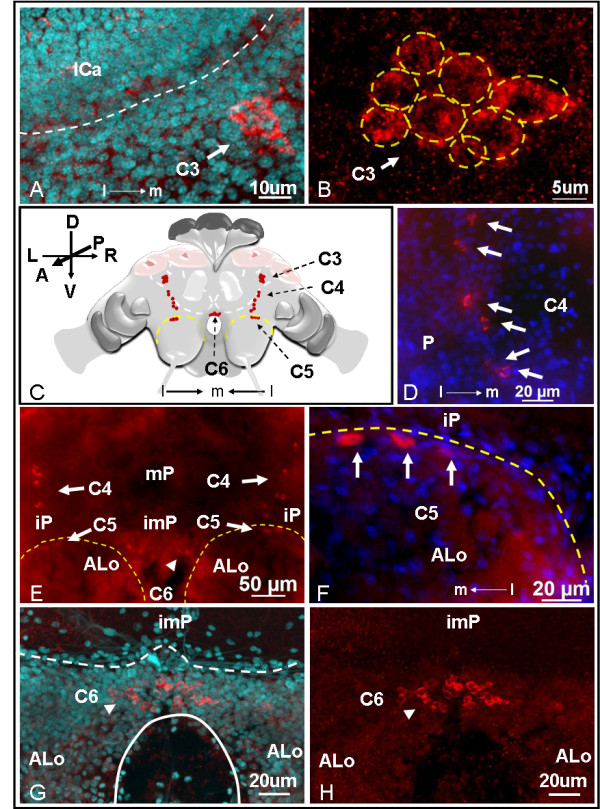
**Distribution of C3 to C6 sNPF receptor immunolabeled clusters observed in the anterior queen brain**. A: Merged confocal image of pictures obtained with red (receptor signal) and cyan (DAPI nuclear staining) channels. B: Image obtained with red channel. In A and B, the cluster C3 (arrow) includes a group of eight cells (B, dashed circles) located on each side of the superior protocerebrum (sP) near the lateral calyces (lCa). C: A schematic shows the relative position of clusters C3 to C6 in the brain. D-H: Whole mount images obtained with Axioimager A1 microscope (nuclei stained in blue with DAPI). D: The cluster C4 includes six cells distributed vertically in the protocerebrum (P). E: The anterior brain view shows the relative position of clusters C4 and C5 (arrows) on each side of the protocerebrum, and the cluster C6 (arrowhead) on the inferior medial protocerebrum (imP). mP, medial protocerebrum; iP, inferior protocerebrum (iP); ALo, antennal lobe. F: A higher magnification image of E showing that the cluster C5 contains three cells horizontally aligned on the superior edge of the antennal lobe which is nearby the inferior protocerebrum. Confocal images G and H: G, merged of red and cyan channels and H, a red channel image. The cluster C6 includes a group of about 30 cells (arrowheads) located at the edge of the inferior medial protocerebrum above the esophageal foramen (G, white solid line). Thickness of stacked confocal images: 8.20 μm for A and B; 18 μm for G and H.

**Figure 5:**
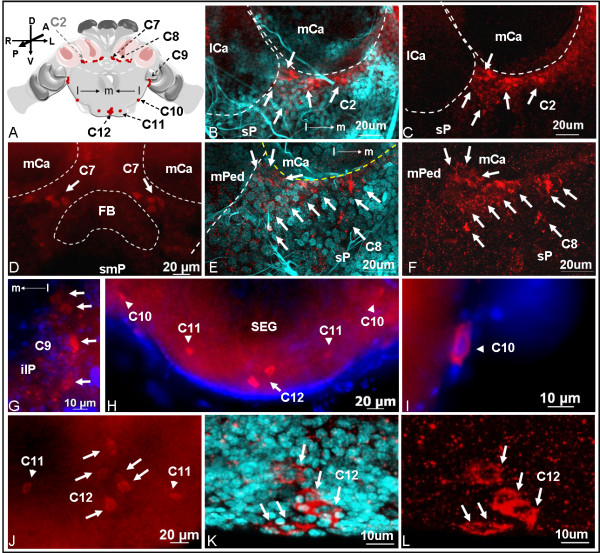
**Distribution of C7 to C12 sNPF receptor immunolabeled cells observed in the posterior brain and SEG**. A: The schematic shows the position of C7-C12. Confocal images obtained with single red channel for receptor signal (C, F, and L) or red merged with cyan (DAPI nuclear stain) (B, E, and K) are shown. B and C: Some cells from the anterior cluster C2 (arrows), located between the mushroom body calyces (mCa and lCa), were also seen from the posterior brain view. D: C7 includes four cells (arrows) located above the fan-shaped body (FB) of the central complex in the superior medial protocerebrum (smP). E and F: C8 is composed of ~11 cells located in the superior protocerebrum (sP) under the medial calyces (mCa). D, and G-J: Whole mount images obtained with Axioimager A1 microscope (nuclei in blue). G: The cluster C9 is composed of four cells (arrows) located in the inferior lateral protocerebrum (ilP). H-J: C10 and C11 are represented by one cell each on each side of the SEG (arrowheads) and C12 is composed of five cells (arrows) centrally located in the SEG. C10-C12 were larger in size (12 μm) and showed strong receptor immunolabeling. I: A C10 single cell is shown. J: C11 and C12 are clearly shown in a ventral view of SEG. K: Stacked confocal images show C12 (five cells, arrows). Thickness of stacked images: B and C, 12 μm; E and F, 14 μm; K and L, 19.6 μm.

In the virgin queen brains and the subesophageal ganglion (SEG), about 164 cells distributed in distinctive cell clusters (C1-C9 and C12) or present as individual cells (C10, C11) were immunolabeled with sNPF receptor antibodies. These cells or clusters were named as C1 to C12, clockwise, beginning from the mid-superior line in the anterior brain view and continuing to the posterior view of brain and the SEG. Clusters C1 to C6 (Figure 2, A) are thus readily seen in the anterior view during brain whole mounts examination, and C7 to C12 (Figure 2, B) are seen in the posterior view while C5, C9 and C12 can also be seen dorsally (Figure 2, C and 2D). Except clusters C1, C6 and C12 which are located centrally, other clusters or cells were bilaterally symmetrical. Most of these neurons are located in or near the important sensory neuropils. Strong sNPF receptor signals were observed in clusters C2, C3, and C12. Three different sizes of cell bodies, small (cells ~5 μm), intermediate (cells ~5-10 μm), and large (cells ~12 μm), were detected with sNPF receptor antibodies. The C6 cluster only contained small cells and C5 contained two large cells and one intermediate size cell, while C10-C12 cells were exclusively large. The rest of the immunostained cells in other clusters were of intermediate size. We did not observe different receptor distribution in the brains of virgin and mated queens. Therefore, results shown in Figures 2, 3, 4, 5 are only from virgin queens. All subsequent brain images in Figures 3, 4, 5 show anterior or posterior brain views with the dorsal side up, unless otherwise mentioned.

Clusters C1 to C6 were observed in the anterior side of the brain (Figures 3 and 4). Cluster C1 (cells ~5-10 μm) included three unpaired cells located in the superior medial protocerebrum (Figure 3, A-D). Cluster C2 (cells ~5-10 μm) is represented by two groups of ~25 cells each, which are located near the lateral calyces (lCa) of the mushroom bodies in the superior protocerebrum (Figure 3, E-L). These cells surrounded the anterior half-side of the lateral pedunculi (lPed) that connects to the lCa (Figure 3, E-H). Out of 25 cells in the cluster C2, seven cells that are located closer to the anterior surface of the lCa are likely to be the lateral neurosecretory cells (Figure 3, E-L, arrowheads). The cluster C7 also is detected in the stacked confocal images (Figure 3, F-G) obtained from the anterior view and will be described further in Figure 5 because it is more clearly visible from the posterior side of the brain.

C3 cells are represented by two groups of eight cells each (cells ~5-10 μm), symmetrically located in the superior protocerebrum (Figure 4, A and 4B). Two groups of six cells (cells ~5-10 μm) named cluster C4 were observed symmetrically located in the anterior protocerebrum, extending vertically between clusters C3 and C5 (Figure 4, D and 4E). Cluster C5 contained three cells horizontally aligned on the superior edge of antennal lobe (Figure 4, E and 4F); stronger signals were detected in the two larger cells. The last cluster of cells observed from the anterior side of the brain was C6 which was composed of a group of ~30 small cells (cells ~5 μm). These cells were centrally located in the inferior medial protocerebrum, right above the esophageal foramen, near the end of the mushroom body median lobe (Figure 4, G and 4H).

C7 to C12 were visible in the posterior side of the brain and SEG (Figure 5). Some cells belonging to the anterior cluster C2 that are located between lCa and medial calyces (mCa) were also visible from the posterior side of the brain (Figure 5, A-C). Cluster C7 (cells ~5-10 μm) includes two groups of four cells symmetrically located above the central complex in the superior medial protocerebrum (Figure 5, D); this brain area is also named the pars intercerebralis in the honey bee. Two groups of about 11 cells (cells ~5-10 μm), each in the superior protocerebrum right below the mCa of the mushroom bodies were named cluster C8 (Figure 5, E and 5F). Cluster C9 (cells ~5-10 μm) includes two groups of 4 cells each located in the inferior lateral protocerebrum; these are likely optical projection neurons (Figure 5, G). In the posterior SEG, large cells (~12 μm) named C10, C11, and C12 were strongly immunolabeled with the anti-sNPF receptor antibodies (Figure 5, H-L). C10 and C11 are represented each by a single cell on each side of the SEG and cluster C12 is centrally located and includes 5 cells.

### The localization of the sNPF receptors in the ovaries of fire ant queens

**Figure 6:**
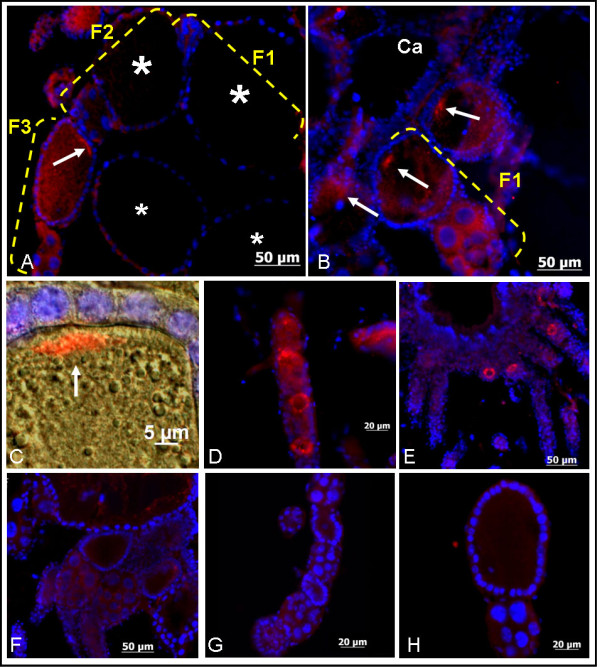
**The immunolocalization analyses of the sNPF receptor in the ovaries of fire ant queens**. Receptor signal was observed in mid- and early-oogenesis oocytes. A: In mated queens in a colony, the sNPF receptor signal was detected in the posterior end of oocytes at the mid-oogenesis stage (arrow, oocyte in follicle number three), but not in oocytes at the late-oogenesis stage in which nurse cells are reduced in size (big stars, oocytes in follicles number 1 and 2). F1, 2 and 3: follicle numbers counted from the calyx (Ca). B: In newly-mated queens (24 h after mating flight), the receptor signals were also detected in a similar stage of developing oocytes as in A. C: A higher magnification merged image of the oocyte posterior end from newly-mated queen ovaries shows a clearly receptor signal (red) in the oocyte membrane, but not in the follicle cells (nuclei in blue, DAPI label). D: The receptor signals were also detected in the early-oogenesis stage oocytes (oocyte size < 20 μm) of mated queens in a colony. E: In virgin queen ovaries, the receptor signals were detected in the early-oogenesis stage oocytes, similar to the mated queen oocytes shown in D. No signal was detected in negative controls using either antigen-preabsorbed antibodies (F and G) or a preimmune antiserum (H).

To discover the possible neurohormone role of the fire ant sNPF peptide(s), we performed the immunolocalization of the sNPF receptor in the ovaries of virgin and mated queens (Figure 6). In the ovaries of both mated queens within a colony (Figure 6, A, arrow) and newly mated queens 24 h after the mating flight (Figure 6, B and 6C, arrows), sNPF receptor signals were detected in the posterior end of oocytes at the mid-oogenesis stage. Results clearly showed that the receptor signals localized in the oocyte membrane, and not in the membrane of follicle cells (Figure 6, C). Such signal was not detected in late-oogenesis stage oocytes in which the nurse cells within the same follicle start to shrink in size (Figure 6, A: F1 and F2, big stars). Mated queens within the colony have more mature eggs in each ovariole than newly-mated queens; therefore, the position of the oocytes with receptor signals depended upon the status of ovary development. The receptor signals were also detected in the early-oogenesis stage oocytes (oocyte size < 20 μm) of the mated (Figure 6, D) and virgin queens (Figure 6, E). Notice that the signal is present in the periphery of the oocytes. No signal was detected using either antigen-preabsorbed antibodies (Figure 6, F and 6G) or a preimmune antiserum (Figure 6, H), as expected. This result supports the function of the sNPF pathway in stimulating oocyte development in addition to roles in the modulation of metabolism, growth, and feeding. To our knowledge, this is the first report that a GPCR may be associated with the oocyte pole.

## Discussion

### Only one form of the sNPF receptor is present in the brains of fire ant queens

Western blot analysis of the fire ant sNPF receptor (Figure 1) showed that only one band at 46.2 kDa was detected in membrane proteins preparations from brains of both virgin and mated queens, corresponding to the predicted receptor Mw of 44.8 kDa [11]. In addition, no differential receptor expression level between virgin and mated queen brains was found, suggesting that receptor abundance in the brains was similar in queens within a colony regardless their insemination status. The only previous study in insects describing the Mw of the sNPF receptor was from the mosquito *An. gambiae *in which two sNPF receptor bands at 50- and 60- kDa were detected in western blot of heads [14]. The authors indicated that the 60 kDa band might represent either another form of the receptor (because there are three predicted start codons in the receptor open reading frame), or alternatively, a post-translationally modified receptor. Unlike this mosquito, there was no evidence of an alternative start codon in the fire ant sNPF receptor cDNA [11].

### Expression of the sNPF receptor in diverse regions of queen brains

In the queen brain and SEG, we identified a total of ~164 sNPF receptor immunolabeled cells, distributed individually (C10, C11) or in clusters (C1-C9, C12). Most of these cells localized in important sensory neuropils such as the mushroom bodies, the antennal lobes, and the subesophageal ganglia. These results suggest that the sNPF receptor is involved in the widespread modulation of neuronal activity and subsequently may affect various physiological processes and behavioral responses in fire ants.

In *Drosophila*, *in situ *hybridization and immunohistochemistry analyses have demonstrated that sNPF transcript or peptide precursor are present in a significant number of neurons in the brain beyond any other neuropeptide found in insects studied so far. In both larvae and adults of *Drosophila*, sNPF peptide precursor was detected in the mushroom bodies, the subesophageal ganglion, and some neurosecretory cells in each brain hemisphere; in addition, this precursor was also present in the fan-shaped body (a substructure of the central complex), the antennal lobe, a few clock neurons, the optic lobes, the tritocerebral neuropil of the adult brain, as well as in the thoracic-abdominal ganglia of the larvae, and some endocrine cells in the larval midgut [31,32,40,41,46-50]. It was suggested that sNPFs are likely to signal locally (no volume transmission) as co-transmitters because, first, these sNPFnergic neurons do not co-express a transcription factor (DIMM) that is present in most of the neurosecretory cells that release amidated peptides, and secondly, these sNPF neurons co-localized with other neurotransmitters [31,51]. Only a few neurons that co-express both sNPF and DIMM in adult flies are likely to act on target cells located at a distance (volume transmission) [1]. There is a paucity of information on the localization of both sNPF and its receptor in other insect orders. Only recently the expression of the sNPF transcript was analyzed in the honey bee worker brain, and found bilaterally only in a few lateral neurosecretory cells (4-6 pairs). This expression pattern contrasts with the wide distribution of the sNPF in neurons of fruit flies described above. Further, the sNPF peptide has not yet been isolated from fire ants and there is no available sNPF peptide sequence identified from the recently released fire ant draft genome [52]. The lack of a cognate peptide has hampered our attempts to prove sNPF receptor functionality in a heterologous expression system. We have identified sNPF receptor immunolabeled cells distributed in many important sensory neuropils in queen brains suggesting that sNPF peptide function may be more complex and perhaps integrative in fire ant queen brains. However, because our immunolabeling did not stain the terminals of the sNPF receptor-labeled neurons we cannot conclude on the nature of these potential networks.

sNPF receptor localization in the protocerebrum of the fire ant brains

In the brain of fire ant queens, we detected three unpaired sNPF receptor immunolabeled cells (cluster C1) located in the superior midline protocerebrum (Figure 3, B-D), where the median neurosecretory cells are located in the honey bee brain [53]. In *Drosophila *larvae, the sNPF receptor was also localized in some of the seven insulin-producing median neurosecretory cells and was verified to act as an upstream regulator of the insulin signaling pathway [33]. In *A. aegypti*, however, insulin-like peptides were detected in two clusters of lateral neurosecretory cells in the dorsal protocerebrum, but not in median neurosecretory cells [54]. It would be interesting to know if the regulatory role of the sNPF signaling pathway on insulin production in different insects is conserved and thus, to investigate by immunohistochemistry if cells in cluster C1 also produce insulin in fire ant queens.

In fire ant queen brains, the majority of the sNPF receptor immunolocalized cells were near the mushroom body. In insects, the mushroom body is the main center for sensory processing, learning and memory, and the integration of other complex behaviors [55]. In Hymenoptera, especially ants, the mushroom bodies are particularly developed and may occupy ~40% of the brain volume [56,57]. The insect brain structure most similar to the fire ant brain is from the honey bee. Both species have large mushroom bodies composed of two calyces (lateral and medial calyces), each with a peduncle which gives rise to median and vertical lobes. The calyx receives input from both the olfactory and visual systems whose neurons synapse with the mushroom body intrinsic neurons, the Kenyon cells. The Kenyon cells consecutively integrate and pass on information within the brain [55]. We have identified several sNPF receptor immunolabeled cells near the mushroom body calyx (clusters C2, C3 and C8) and near the (output) end of the mushroom body median lobe (cluster C6) in the queen brains. Based on the comparison of the location of cluster C2 cells with the position of lateral neurosecretory cells in the brains of honey bees [53], it is possible that some of the C2 cells might belong to lateral neurosecretory cells (Figure 3, E-L, arrowheads). Other neurons in clusters C2 (Figure 3, E-H, arrows) and C8 (Figure 5, E and 5F) were similar in location to the Clawed II Kenyon cells found in the honey bee which are perikarya lying outside the calyx [58,59]. We therefore hypothesize that the sNPF peptide regulates Kenyon cell function in fire ant queens.

Cells in the cluster C3 (Figure 4, A-B) between the medium and lateral calyces of the mushroom bodies are tightly group together in the superior protocerebrum, reminiscent in location and aspect to the group 4 FMRFamide-like immunoreactive cells identified in the honey bee brain [60].

The cluster C6 located in the inferior medial protocerebrum is very close to the end of mushroom body median lobe (Figure 4, G and 4H). These C6 cells may represent the targets of axon outputs of sNPFnergic Kenyon cells in the queen brains. In *Drosophila*, the sNPF peptides are the only neuroactive substance that has been clearly identified in large subpopulations of intrinsic Kenyon cells, and strong sNPF signals are detected in the end (output) of median and vertical lobes in the mushroom bodies [31,48]. However, in fire ant brains, we did not detect other receptor signals near the end of the vertical lobe. This might be due to the largely variant number of Kenyon cells between insect species. For example, in the honey bee each mushroom body has ~170,000 Kenyon cells [61], much more than in *Drosophila *(~2,500 Kenyon cells) [62]. As a result, the expression of sNPF peptide in the Kenyon cells may also be different between insect orders.

The central complex has been proposed as a higher center for locomotor control that regulates several aspects of walking and flying behaviors [63]. The cluster C7 localized above the fan-shaped body of the central complex (Figure 5, D) is located in the same area as the cluster G4d octopaminergic neurons in the honey bee brain [64]. In honey bees, octopamine treatment caused increased flying and reduced walking behaviors [65]. In *Drosophila*, octopamine is also associated with locomotor behaviors [66]. Interestingly, a recent study showed that *Drosophila *sNPF peptides expressed in the fan-shaped body of the central complex were associated to the fine tuning of locomotor activity [41]. Female flies with reduced sNPF peptide expression in the fan-shaped body increased their walking distance and their mean walking speed. Thus, the relationship between the sNPF receptor and locomotor activity (which is regulated by sNPF and octopamine in *Drosophila *or other insects) deserves further investigation in fire ants. If these were related, it will contribute to the understanding of worker foraging behavior, expansion of established colonies and of regulation of queen mating flights. Knowledge of these mechanisms that contribute to colony growth and dispersal may open the possibility of controlling this invasive pest.

In the optic lobes, we detected sNPF receptor signals in four cell bodies (cluster C9) in the inferior lateral protocerebrum in the fire ant (Figure 5, G). No other sNPF receptor signal above background staining was obtained in the optic lobe. In the desert ant, *Cataglyphis albicans*, the optical projection neurons located in the similar inferior lateral protocerebrum region have their dendrites extended to the optic lobes, and their axons projected into the mushroom bodies [67]. Due to the similarity of location, we hypothesize that the C9 sNPF-immunoreactive neurons correspond to the optical projection neurons observed in *Cataglyphis albicans*. In the brain of adult flies, both the sNPF transcripts and peptides are localized in the optic lobes [31,32].

### sNPF receptor localization in the antennal lobe of the fire ant brains

The antennal lobe is the primary olfactory neuropil. In the antennal lobe, the axons of olfactory receptor neurons from the antennae and the maxillary palps invade into the glomeruli where they synapse with dendrites from interneurons. In queen brains, the cluster C5 cells were horizontally aligned on the superior edge of the antennal lobe. There are two large cells and one intermediate size cell in the cluster C5 (Figure 4, F). It is possible that these C5 cells act in functionally different ways, and belong to different types of interneurons. In insects, three types of interneurons have been distinguished in the antennal lobe: 1) local interneurons that locally interconnect with glomeruli; 2) projection neurons that innervate single or several glomeruli and project axons through antennocerebral tracts into the protocerebrum; 3) centrifugal neurons that are a diverse grouping of neurons with axons projected into the antennal lobe and dendrites reaching to other areas of the nervous system [68]. Cells in the cluster C5 could be local and/or projection interneurons. In *Drosophila*, the sNPF precursor was immunolabeled in axons of the olfactory receptor neurons (ORNs) projecting from the antennae and the maxillary palps which terminate in 13 glomeruli, with the glomerulus DL3 being particularly strongly stained [49]. It has been shown that the sNPF peptide is involved in the modulation of olfactory processing based on the hunger state of the individual fly [69]. In the fly's anterior dorsal antennal lobes, the projection neurons have their dendrites invade into a subset of glomeruli, including sNPF-labeled glomeruli, and have their axons projected through the inner antennocerebral tract (iACT) to the mushroom body and the lateral horn of the protocerebrum [70]. In the fire ant queens, if sNPF peptide(s) were also expressed in antennal ORNs, it is possible that the chemosensory and olfactory information transmitted through these peptides might be delivered to the projection interneurons in the cluster C5 and be further transmitted for integration to the higher brain centers.

### sNPF receptor localization in the subesophageal ganglion (SEG)

The SEG is the primary center for controlling insect mouth parts. Important modulatory neurons, such as ventral unpaired median (VUM) neurons have their cell bodies in the SEG [71]. In the honey bee, octopaminergic VUM cells innervate the neuropils of the SEG, the mandibulla, the antennal lobes, the mushroom bodies, and some other parts of the brain [72]. The sNPF receptor immunolabeled cells C10, C11, and cluster C12 in the SEG of the fire ant (Figure 5, H-L) were in similar location as the octopaminergic neurons G6b, LV (the lateral ventral), and VUM in the SEG of the honey bee, respectively [64]. In honey bee workers, octopamine modulates diverse behaviors including locomotor activity, dance, division of labor, foraging, hygiene, defense, and nestmate recognition [65,73-80]. In fire ant workers, it was also found that octopamine levels in the brain affect nestmate recognition acuity [81]. However, the function of octopamine in the queen brain of both insects is still unknown. It will be of interest to test if these sNPF receptor immunolabeled cells in the fire ant SEG are involved in regulation of feeding and/or are octopaminergic neurons.

### The expression of the sNPF receptor in fire ant ovaries

Receptors that influence ovarian growth are critical for egg-laying species, as they play a role in oocyte development regulation. Recently some GPCRs involved in meiosis arrest and fertility in vertebrate oocytes were identified [82-85]; yet to our knowledge, no GPCR function has been found associated to the polarity of oocytes in insects. Only few receptors from other receptor superfamilies are involved in the follicle polarity, such as follicle cell-bound EGF receptor involved in the transduction of oocyte anterior/posterior polarity, and Frizzled, involved in the planar polarity signaling [86,87]. In fire ants, the sNPF receptor signal detected in the posterior end of the oocytes during mid-oogenesis from both mated queens (Figure 6, A) and newly-mated queens (Figure 6, B and 6C) opens the possibility that the sNPF receptor may be involved in oocyte polarity. To our knowledge, this is the first report of GPCR that has been found associated with an oocyte pole in insects. We also detected the sNPF receptor signals in the periphery of early-oogenesis stage oocytes from both mated and virgin queens, most likely in the plasma membrane, indicating the sNPF receptor may participate in the regulation of initial oocyte growth (Figure 6, D and 6E).

However, we did not detect putative receptor bands in membrane preparations from virgin queen ovaries by western blot (Fig. 1, B, lane 3). This might be due to a limited number of developing oocytes with corresponding low receptor expression levels in the virgin queens which prevented receptor protein detection. In the past we had a similar experience with respect to the lack of detection in western blots from virgin queen ovaries with the antibody against the vitellogenin receptor which labeled the ovaries in immunohistochemistry but failed to detect the protein in western blots (Lu and Pietrantonio, unpublished). In addition, because two weak bands were also detected by western blot in the 60-75 kDa range in both virgin and mated queen ovaries (Fig. 1, B, lanes 3, 4), we are not ruling out the possibility that the fluorescence signals in the early-oogenesis stage oocytes could represent non-specific antibody binding to those two higher size bands. If this is the case, then only the signal in the oocyte posterior end in the mated queen would be specific sNPF receptor signal.

Different results on sNPF receptor expression were obtained in the mosquito. In *An. gambiae *sNPF receptor immunoblot analysis showed that the receptor protein was not detectable in the ovaries of non-blood fed females [14]. Based on our results, we concluded that this might be due to the fact that the females were not blood fed and therefore the ovaries were underdeveloped. In *Drosophila*, sNPF receptor transcripts were detected in the ovaries of females of unknown age, and the expression level was higher than in the head and body parts [43]. However, in the Flyatlas transcriptome data bank which utilized mRNA from 7 day-old adult fly http://www.flyatlas.org/, the sNPF receptor mRNA level in the ovaries was very low when compared to whole flies [88]. All together, it seems that the level of sNPF receptor protein abundance in the ovaries depends upon the level of the ovary development, mated status, the age of the insects and is perhaps different between solitary and social insects.

## Conclusions

We presented the first comprehensive histochemical analysis of the distribution of the sNPF receptor in the adult insect brain. This knowledge is especially important for research on social insects which display complex social and learning behaviors. The sNPF receptor signals present in several neuropils in fire ant queen brain and SEG might link the receptor signaling pathway to behaviors such as foraging, learning, and food consumption. The localization of the sNPF receptor will allow the testing of the potential regulatory role of this pathway in insulin-producing or octopaminergic cells. In addition, the localization of the sNPF receptor in the developing oocyte points to a direct, potentially novel effect of sNPF on the insect ovary. Still, several questions remain to be answered to fully understand the role of sNPF pathway in neuron circuits, in the endocrine control of reproduction, and in other, yet unknown functions. These and other studies will be facilitated by the development of novel specific antibodies and neuronal markers as the annotation of the fire ant genome is completed.

## Methods

### Insects

Polygyne (multiple-queens) colonies of *S. invicta *were obtained from the Fire Ant Research Laboratory in the Department of Entomology (Texas A&M University, College Station, TX, USA) and maintained as previous described [89]. All colonies were screened for absence of microsporidium (*Kneallhazia *(=*Thelohania*) *solenopsae*) spores infection. Virgin queens (alates) and mated queens (de-alates) were collected from multiple polygyne colonies. Newly mated queens were collected from the field after mating flights; queens can be abundantly found at around 3-4 p.m. Queens were brought to the laboratory and maintained at 27°C in glass tubes which acted as humidity chambers by half-filling them with water and cotton. Ovaries were dissected 24 h after collection. During dissection, successfully mated queens were identified by observing an inseminated large and white spermatheca; only tissues from these inseminated queens were used.

### Antibodies

To determine the antigenic regions of fire ant sNPF receptor (GenBank: DQ026281) for anti-peptide antibody production, the hydrophilicity and antigenicity profiles of sNPF receptor amino acid sequence were analyzed using DNASTAR and ExPAsy software. Additionally, the N-glycosylation and phosphorylation sites were avoided in the selection for the antigenic region. Due to a very short N-terminal sequence present in the receptor before the first transmembrane region (total 23 residues), only one amino acid region located toward the receptor C-terminus encompassing residues 331 to 347 of sequence "CRGDKIDNGNNTMQETL" was selected for antibody production. Polyclonal anti-peptide antibodies were developed in New Zealand female rabbits by Pacific Immunology (CA, USA) and affinity purified. The synthetic peptide was conjugated with keyhole limpet hemocyanin (KLH) for antibody production. After purification, the specificity of the antibodies was verified by ELISA (tested by Pacific Immunology, CA) and western blot.

### Membrane preparations and western blot analysis

Membrane preparations for western blot analyses were performed as described previously with minor modifications [90]. Briefly, brains (with SEG) and the postpharyngeal glands from about 200 virgin queen heads were dissected in phosphate buffered saline (PBS). In addition, brains (with SEG) and ovaries from about 200 virgin queens and 200 mated queens were also dissected in PBS. These tissues were homogenized in cold buffer A (25 mM Tris⁄HCl, pH 7.5, 1 mM EDTA, 1 mM EGTA, 1 mM dithiothreitol) with Complete Protease Inhibitor Cocktail^® ^(ROCHE) and centrifuged at 10,000 g for 5 min. The supernatants were collected and centrifuged at 100,000 g (SW28 rotor, Beckman LE80K) for 1 h at 4°C. After ultracentrifugation, the pellets were re-suspended in 200 μl cold buffer B (50 mM Tris ⁄ HCl, pH 7.5, 2 mM CaCl_2_) with protease inhibitors and stored at -80°C.

For western blot analysis, membrane proteins (100 μg) were separated on SDS-PAGE (10% gel, Bio-Rad) and transferred to PVDF membranes (Millipore). Membranes were blocked 1 h at room temperature in blocking solution [5% non-fat milk in TBST (10 mM Tris base, 140 mM NaCl, 0.1% Tween-20, pH 7.4)] and incubated overnight with rabbit anti-sNPF receptor antibodies (1:500) in blocking solution at 4°C. Pre-absorbed anti-sNPF receptor antibodies and pre-immune serum were used as negative controls. Pre-absorbed antibodies were produced by incubating anti-sNPF receptor antibodies (4 μg in 10 ml blocking solution) with peptide antigen (500 μg) overnight at 4°C the day previous to their use. After 3 × 10 min washes with TBST, the membrane was then incubated with a of HRP-conjugated goat anti-rabbit IgG antibody (1:40,000) for 1 h. After the same wash steps, the membrane was visualized by the Enhanced Chemiluminescence System™ (Pierce) on film (Kodak).

### Brain and SEG whole mount immunofluorescence analysis

Whole mount immunofluorescence was performed as previously described with modifications [91]. Brains of both virgin and mated queens were dissected in PBS and transferred into freshly prepared 4% paraformaldehyde in PBS for 2 h at 4°C for fixation. All subsequent steps were carried out with slow agitation. The fixatives were removed by 3 × 10 min washes with 70% ethanol, on ice. After rinsed in PBST (PBS with 0.1% Tween) 2 × 5 min at room temperature, tissues were incubated in 12 μg/ml protease K (Sigma-Aldrich) in PBS for 10 min at room temperature. Tissues were then rinsed in PBST 2 × 5 min and then blocked in PBST with 10% normal goat serum (NGS) (Sigma-Aldrich) for 24 h at 4°C. After blocking, tissues were incubated with primary antibody for 48 h at 4°C. The primary antibodies diluted in PBST-2% NGS included the anti-sNPF receptor antibodies (diluted 1:100). For negative controls, anti-sNPF receptor antibodies (1:100 dilution) pre-absorbed with the antigen peptide, and pre-immune serum from rabbit (1:1000 dilution) were used as the primary antibody. Anti-sNPF receptor antibodies (4 μg in 1 ml PBST-2% NGS) were pre-absorbed with 500 μg of antigen peptide overnight at 4°C incubation one day before used.

After incubated with primary antibodies, tissues were washed 4 × 20 min in PBST and incubated in a 1:200 dilution of Alexa Fluor 546 goat anti-rabbit IgG (Invitrogen™) overnight at 4°C. Tissues were then washed 6 × 30 min in PBST and mounted on slides with Vectashield™-DAPI (Vector, Burlingame, CA, USA) for nuclear staining. Results were observed under a Carl Zeiss Axioimager A1 microscope. Images were obtained with an AxioCam MRc color camera (Carl Zeiss) and analyzed with axiovision program (Carl Zeiss). Confocal images were taken using FV1000 Confocal microscope (Olympus) in Microscopy and Imaging Center (TAMU) and images were analyzed with Olympus FV10-ASW program (Olympus).

Naming of the brain structures was following previous articles on honey bee [59]. Orientation of neuronal structures was given according to the body axis.

### Ovary immunofluorescence analysis

Ovaries of virgin and mated queens within colonies, and ovaries of field-collected newly-mated queens (24 h after mating flight) were dissected in PBS and fixed in 4% paraformaldehyde for 4 h at 4°C. The procedures of ovary paraffin wax blocks, de-waxing and rehydration of ovary sections (12 μm), and ovary immunofluorescence labeling were as previously described [90]. Sections were blocked with blocking solution (5% goat serum in PBS with 0.05% Triton X-100) 1 h at room temperature and then incubated overnight in a humid chamber at 4°C with the anti-sNPF receptor antibodies (1:1000 dilution) in the blocking solution. Negative control sections were also incubated overnight with the pre-absorbed anti-sNPF receptor antibodies (1:1000 dilution) or the pre-immune sera (1:500 dilution) in blocking solutions. The preabsorbed anti-sNPF receptor antibodies were produced as described above.

## List of abbreviations

sNPF: short neuropeptide F; CNS: central nervous system; mCa: medial calyces; lCa: lateral calyces; sP, slP, smP, iP, ilP, imP: the superior, the superior lateral, superior medial, inferior, inferior lateral, and inferior medial protocerebrum, respectively; SEG: subesophageal ganglion.

## Authors' contributions

H.-L. Lu designed and performed research. P.V. Pietrantonio designed research. H.-L. Lu and P.V.Pietrantonio analyzed result and wrote the paper. All authors read and approved the final manuscript.
